# Cutaneous angiosarcoma metastatic to small bowel with nodal involvement 

**Published:** 2016

**Authors:** Vidya A Fleetwood, Jamie C Harris, Minh B Luu

**Affiliations:** *Department of General Surgery, Rush University Medical Center, Chicago, Illinois, USA *

**Keywords:** Small bowel cancer, Cancer metastasis, Angiosarcoma

## Abstract

A 77-year-old male with a history of metastatic scalp angiosarcoma presented with intractable gastrointestinal bleeding from a jejunal mass detected on capsule endoscopy. He underwent laparoscopic-assisted resection of the mass. Intraoperatively, an isolated small bowel mass with bulky lymphadenopathy was seen and resected en bloc. Pathology showed a 6.8cm high-grade metastatic angiosarcoma with nodal involvement and negative margins. Angiosarcoma is a sarcoma with a grim prognosis. The incidence is 2% of all soft tissue sarcomas; cutaneous lesions comprise 27% of manifestations and usually appear on head and neck. Risk factors include lymphedema, neurofibromatosis, vinyl chloride, arsenic, and anabolic steroids. Overall 5-year survival is 30-35% and is higher in patients younger than 60, those without metastasis, tumors less than 5 cm, and favorable histology. Angiosarcoma metastasis to small bowel is rare but nodal involvement is even more unusual, reported only three times in the literature. This case is the first with nodal involvement to present at a resectable stage. To diagnose disease when still at a resectable stage, a high index of suspicion must be maintained with any gastrointestinal symptoms in a patient with a history of angiosarcoma. Laparoscopic-assisted resection is safe for the resection of small bowel angiosarcoma.

## Case Presentation

 A 77-year-old male presented to our clinic with gastrointestinal bleeding. He had recently been admitted for melena and iron deficiency anemia, requiring multiple transfusions. Workup was benign, with EGD and colonoscopy significant only for non-bleeding internal hemorrhoids. His medical history was significant for angiosarcoma of the scalp which had been excised and radiated; he had had metastases to his neck and lungs resected and had undergone radical neck dissection and radiation. He had refused systemic chemotherapy. Laboratory results on presentation were significant for hemoglobin of 7.3 g/dL (normal range 13.5-17.55) and were otherwise unremarkable. Abdominal exam was benign with no masses or tenderness.

**Figure 1:**
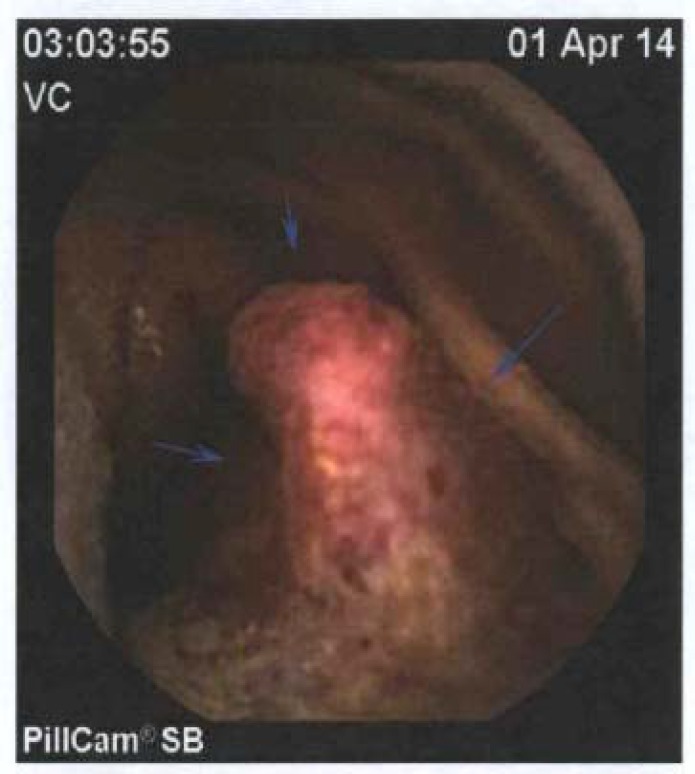
Video capsule endoscopic imaging of small bowel mass

 His endoscopy showed no culprit lesions. He was referred for video capsule study, which revealed a large mass in the proximal small bowel with small amount of blood obscuring lesion (Figure 1). With otherwise normal workup, this was presumed to be the source of gastrointestinal bleeding. He was transfused and taken to the operating room for hand-assisted laparoscopic small bowel resection. 

**Figure 2:**
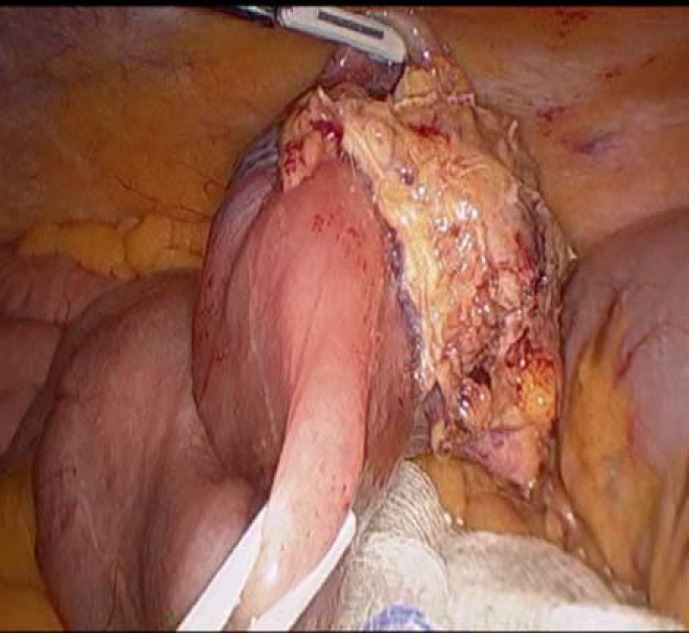
Small bowel mass with mesenteric nodal involvement

After entering abdomen, the small bowel was run from the ligament of Treitz until the lesion was identified. The mass was found in mid-jejunum, protruding from small bowel lumen and adherent to the small bowel mesentery (Figure 2). 

Involved jejunum was exteriorized for resection. Upon inspection, gross lymphadenopathy was noted to the root of the mesentery; this was divided without vascular compromise. The small bowel was resected for 10cm gross margins. The intestine was examined to rule out satellite lesions and the operation was concluded. Postoperative course was notable only for a prolonged ileus; no further bleeding episodes were noted during the hospital stay. 

 Pathology returned noting 6.8cm mass positive for high-grade metastatic angiosarcoma. Margins were microscopically negative. Three of four resected nodes were positive for angiosarcoma. 

 The patient has not experienced any bleeding episodes since resection. 

## Discussion

Angiosarcoma is a vascular sarcoma characterized by endothelial origin, local aggression, distant metastases, and a grim prognosis (1). The overall incidence is poorly cited but is thought to be 2% of all soft tissue sarcomas (1). Most lesions are cutaneous and develop on the head and neck, breast, or extremities; metastases are chiefly spread hematogenously to the lung (1). 

 Gastrointestinal angiosarcoma, primary or metastatic, is exceedingly rare. In a literature review by Qingqiang et al., (2) a total of 40 patients with angiosarcoma presenting primarily in the small bowel are cited in the literature up to 2013. Of these patients, eleven presented with hematochezia or melena, but the remainder presented with occult bleeding, abdominal pain alone, obstruction, or acute abdomen. 

Rates of metastasis to the small bowel are even more unusual; our literature review (using search terms “angiosarcoma”, “metastasis”, and “small intestine” separately on PubMed, limited to English language and humans) revealed only six cases in the English literature (2-6). Five cases presented with bleeding (4) and one with acute abdomen (5). 

 The nodal metastasis we saw intraoperatively is atypical of angiosarcoma. Our review revealed only three cases with gastrointestinal nodal metastasis. All cases presented with an advanced stage of disease, including: multiple unresectable lesions (2), chylous ascites secondary to lymphatic obstruction by tumor (6), and acute abdomen secondary to intestinal perforation (2). 

There is insufficient information, either in cutaneous or visceral angiosarcoma, to support the role of surgical nodal debulking; however, the presence of nodal metastasis certainly warrants a referral to medical oncology and the serious consideration of systemic treatment if the patient survives the initial operation. 

Although rare, the presence of these lesions in the literature and their late detection and poor survival suggest that a high index of suspicion must be maintained in patients with a primary angiosarcoma who present with gastrointestinal symptoms, especially those of bleeding or pain.

The presence of GI metastasis in patients with angiosarcoma is rare, but has been defined in the literature. Any new presentation of GI bleeding in a patient with known angiosarcoma or with suspicious skin lesions should be worked up aggressively and early, as most GI metastases present at an advanced stage. We suggest a full evaluation, including EGD and colonoscopy early in the clinical course with VCE included regardless of findings, given the incidence of these tumors in the small intestine and the relatively high rates of multifocal disease. Any patient who presents with isolated angiosarcoma of the intestine should also undergo a full skin exam at the time of presentation to rule out a cutaneous source. With vigilance, we can detect these lesions earlier in their course, at a time when they are more amenable to resection and ultimately, to survival.
